# Human Muscle Protein Synthesis Rates after Intake of Hydrolyzed Porcine-Derived and Cows’ Milk Whey Proteins—A Randomized Controlled Trial

**DOI:** 10.3390/nu11050989

**Published:** 2019-04-30

**Authors:** Line Q. Bendtsen, Tanja K. Thorning, Søren Reitelseder, Christian Ritz, Erik T. Hansen, Gerrit van Hall, Arne Astrup, Anders Sjödin, Lars Holm

**Affiliations:** 1 The Department of Nutrition, Exercise and Sports, Faculty of Science, University of Copenhagen, DK-1958 Frederiksberg C, Denmark; linequist@gmail.com (L.Q.B.); tkthorning@gmail.com (T.K.T.); ritz@nexs.ku.dk (C.R.); ast@nexs.ku.dk (A.A.); amsj@nexs.ku.dk (A.S.); 2 Institute of Sports Medicine Copenhagen, Department of Ortopaedic Surgery M, Bispebjerg Hospital, DK-2400 Copenhagen NV, Denmark; s.reitelseder@gmail.com; 3 Department of Biomedical Sciences, Faculty of Health and Medical Sciences, University of Copenhagen, DK-2200 Copenhagen N, Denmark; gerrit.van.hall@regionh.dk; 4 Danish Crown Ingredients, DK-1711 Copenhagen V, Denmark; eth@dat-schaub.dk; 5 Clinical Metabolomics Core Facility, Clinical Biochemistry, Rigshospitalet, DK-2200 Copenhagen N, Denmark; 6 School of Sport, Exercise and Rehabilitation Sciences, University of Birmingham, Birmingham B15 2TT, UK

**Keywords:** dietary proteins, porcine proteins, muscle protein synthesis, amino acids, FSR

## Abstract

Background: Whey protein has been shown to be one of the best proteins to stimulate muscle protein synthesis rate (MPS), but other high quality proteins, e.g., animal/porcine-derived, could have similar effects. Objective: To investigate the effects of hydrolyzed porcine proteins from blood (HPB) and muscle (HPM), in comparison to hydrolyzed whey protein (HW), on MPS after intake of 15 g alone or 30 g protein as part of a mixed meal. We hypothesized that the postprandial MPS would be similar for porcine proteins and whey protein. Design: Eighteen men (mean ± SD age: 24 ± 1 year; BMI: 21.7 ± 0.4 kg/m^2^) participated in the randomized, double-blind, three-way cross-over study. Subjects consumed the three test products (HPB, HPM and HW) in a random order in two servings at each test day. Serving 1 consisted of a drink with 15 g protein and serving 2 of a drink with 30 g protein together with a mixed meal. A flood-primed continuous infusion of (*ring*-^13^C_6_) phenylalanine was performed and muscle biopsies, blood and urine samples were collected for determination of MPS, muscle free leucine, plasma amino acid concentrations and urea excretion. Results: There were no statistical differences between the MPS measured after consuming 15 g protein alone or 30 g with a mixed meal (*p* = 0.53) of HPB (0.048 ± 0.007 vs. 0.049 ± 0.008%/h, resp.), HPM (0.063 ± 0.011 vs. 0.062 ± 0.011 %/h, resp.) and HW (0.058 ± 0.007 vs. 0.071 ± 0.013%/h, resp.). However, the impact of protein type on MPS reached statistical tendency (HPB vs. HPM (*p* = 0.093) and HPB vs. HW (*p* = 0.067)) with no difference between HPM and HW (*p* = 0.88). Plasma leucine, branched-chain, essential and total amino acids were generally higher for HPB and HW than HPM (*p* < 0.01), which reflected their content in the proteins. Muscle-free leucine was higher for HPB than HW and HPM (*p* < 0.05). Conclusion: Hydrolyzed porcine proteins from blood and muscle resulted in an MPS similar to that of HW, although with a trend for porcine blood proteins to be inferior to muscle proteins and whey. Consequently, these porcine-derived muscle proteins can be used similarly to whey protein to support maintenance of skeletal muscle as part of supplements and ingredients in foods.

## 1. Introduction

Daily provision of essential amino acids (EAA) through intake of dietary protein is a necessary composite of an adequate daily energy intake to support overall *de novo* protein synthesis, both in the context of this paper and for the general maintenance of muscle mass [1,2]. The peripheral availability of EAA, often reflected by the postprandial elevation of circulating amino acid, is dependent on (i) the quantity of ingested protein [3], and (ii) the relative content of the EAA in the protein and even specific amino acids [4], as well as (iii) the digestibility and absorption rate of the protein [5,6], with the latter two being components for evaluating the quality of a protein source [7]. However, both protein amount and energy content are decisive for the overall net protein balance in response to a meal [8]. Whey proteins are complete in amino acid composition and appear superior to e.g., casein in stimulating MPS in the immediate postprandial hours [9,10,11]. A quick digestibility and hence availability of constituent amino acids in the circulation is important for the postprandial stimulation of muscle protein synthesis (MPS) [12]. Hence, whey protein, being high in EAA and rapidly absorbed, appears as one of the most anabolic proteins [13]. Many animal proteins are also complete proteins containing all EAA and therefore, they are also of interest. Meat proteins have been shown to stimulate MPS [14,15,16,17]. Burd et al. [14] investigated the increase in MPS following consumption of 30 g protein from skimmed milk or minced beef post exercise and found that milk resulted in a higher MPS compared to beef in the early phase (0–2 h post exercise), most likely due from the faster appearance of leucine after milk intake, while there was no difference on MPS between proteins in the later phase (2–5 h post exercise) where the amino acid availability was sufficient to maintain an elevated MPS after both intakes. Hence, if animal-derived protein can be made more readily accessible after consumption, the stimulatory capacity on MPS may become as good as that of whey protein (the Golden Standard) and hence, make up an alternative protein source. Porcine meat is widely consumed in Europe and Asia and residual proteins from such slaughter animals are highly available [18]. Such proteins from meat side-streams and slaughter animal residuals make up its own business and if they could be used for human feed would add major value of the slaughter animals. Further, taste, solubility and heating tolerance are characteristics related to specific proteins and the slaughter animal-derived ingredients will widen the variability of foods and applications for the benefit of both industry and consumers. Proving a high anabolic potential of such animal-derived protein ingredients will therefore provide arguments for an added-value of foods, a decreased waste generation, and support sustainability. Therefore, this study is aimed at investigating the anabolic potential of such residual porcine protein hydrolysate ingredients and comparing it with high quality whey protein hydrolysate with the purpose of investigating novel ingredients for improvement of protein quality of new foods for human nutrition. 

Hence, we studied the postprandial MPS response after intake of 15 g protein hydrolysates derived from porcine blood or muscle and compared that to the response after intake of 15 g hydrolyzed whey protein. Further, to explore any interaction with energy in a mixed meal on MPS, we subsequently provided a larger dose of 30 g protein in a drink served with a mixed meal. We hypothesized that the postprandial increase in MPS would be similar for the porcine-derived proteins and whey protein. 

## 2. Subjects and Methods

### 2.1. Subjects

Twenty-two normal weight (BMI 18.5–25.0 kg/m^2^) men aged 22–40 years were recruited for the study by advertisements at webpages from August 2015 to April 2016. All subjects participated in a physical screening and 18 subjects were selected for the study based on the following criteria: healthy, non-vegetarian, non-smokers, no drug abuse, low-to moderate alcohol consumption, no regular engagement in cardio or strenuous physical training, no use of protein supplements, no use of medication, no blood donation three months prior to the study, no participation in other clinical studies four weeks prior to the study and hemoglobin >8.0 mmol/L. Moreover, all subjects were weight stable at inclusion (±3 kg within the previous three months) and were instructed not to change their dietary pattern or physical activity level throughout the study period. 

The study was conducted according to the guidelines laid down in the declaration of Helsinki II and all procedures involving human subjects were approved by the Danish National Committee on Health Research Ethics (journal number: H-15003581). Written informed consent was obtained from all subjects after verbal and written information about the study procedures. The trial was registered on clinicaltrials.gov as NCT02477410.

### 2.2. Physical Screening

The physical examination conducted at the screening visit included measurements of body weight, height and body composition. Body weight was recorded to the nearest 0.1 kg (Lindeltronic 8000S, Lindells, Sweden) and height was measured with a wall-mounted stadiometer to the nearest 0.5 cm. Body composition was determined by Dual-energy X-ray absorptiometry (DXA) (Lunar Radiation Co., Madison, WI, USA). All measurements were performed with subjects wearing only underwear and after a 12 h fast. 

### 2.3. Experimental Design

The study was a randomized, double-blind (subjects and researchers), three-way cross-over study, where subjects in a random order received the following treatments (1) hydrolyzed porcine blood protein (HPB, Danish Crown Ingredients, Copenhagen V, Denmark), (2) hydrolyzed porcine muscle protein (HPM, Danish Crown Ingredients, Copenhagen V, Denmark) and (3) hydrolyzed whey protein (HW, Lacprodan, Arla Foods Ingredients group P/S, Viby Jylland, Denmark). The randomization was done by a technician not taking part in the experimental trials.

**Figure 1:**
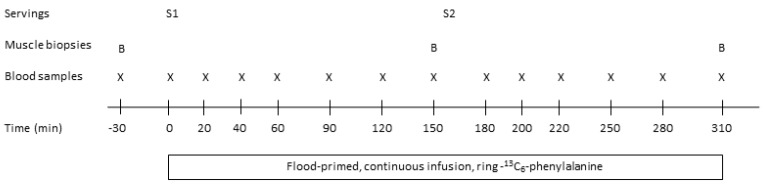
Experimental design. All 18 subjects completed three identical visits during the study period. B, muscle biopsy, S1, serving 1 (15 g protein); S2, serving 2 (30 g protein served with a mixed meal). Time is given in minutes (min) around time point zero (0) where the tracer infusion (flood-primed, continuous infusion) was started and S1 was provided.

All subjects completed three identical intervention visits that were separated by at least two weeks (Figure 1). The night before the intervention visits subjects were asked to consume a standardized meal at home no later than 20.00 h. The meal was provided as freeze product beforehand and subjects were asked to consume the entire meal. After fasting for approximately 12 h (0.5 L water was allowed), subjects arrived by public transportation or car at the Department of Nutrition, Exercise and Sports, University of Copenhagen at 7.30 h.

Upon arrival urine was collected to ensure an empty bladder and body weight was recorded. Moreover, a venflon catheter (Venflon^TM^ Pro I.V. Cannula, Becton Dickinson, Mountain View, CA, USA) was placed in an antecubital vein for infusion of [*ring*-^13^C_6_] phenylalanine (Cambridge Isotopes Laboratories, Andover, MA, USA) and a second catheter was placed in a superficial hand vein in the opposite arm for repeated blood sampling using the heated hand box technique [19]. A background blood sample was drawn and in trial two and three also a muscle biopsy from *vastus lateralis* was obtained. Thereafter, an infusion with [*ring*-^13^C_6_] phenylalanine (Cambridge Isotopes Laboratories, Andover, MA) was initiated. First, a flood-primed infusion (1320 mg unlabeled and 105.6 mg labelled [*ring*-^13^C_6_] phenylalanine in 100 mL suspension) was given over 2–5 min, followed by a continuous infusion of [*ring*-^13^C_6_]phenylalanine (~0.855 mg × fat free mass (kg)^−1^ × hour^−1^) for five hours to maintain isotopic steady state at an estimated tracer-to-tracee ratio (TTR) of 8% [20]. This infusion protocol allowed us to save time (approximately 60–120 min) which is required before isotopic steady state is achieved during a classic primed-continuous amino acid tracer infusion. 

Hereafter, at 8.30 h (time 0 min), subjects were served a protein drink for breakfast (serving 1: 15 g protein) together with 150 mL water. Blood samples were then drawn after 20, 40, 60, 90, 120 and 150 min. After 150 min the second muscle biopsy was taken from *vastus lateralis* and at 160 min a second protein serving was provided (serving 2: 30 g protein) with 200 mL. of water together with toast bread (see section Protein servings). Blood samples were then drawn at the following times 180, 200, 220, 250, 280 and 310 min. At time 310 min the third muscle biopsy was taken from *vastus lateralis* and the infusion with [*ring*-^13^C_6_] phenylalanine was stopped. Urine samples were collected from 0–160 min (serving 1) and from 160–310 min (serving 2) to determine postprandial urea excretion.

### 2.4. Protein Servings

Subjects were provided two protein servings at each intervention visit. The two protein servings included a protein drink containing one of the three proteins of interest; HPB, HPM or HWP. Energy density, macronutrient composition and fiber content were similar between the three dietary treatments. Serving 1 consisted of a drink containing 15 g hydrolyzed protein dissolved in 100 mL water added 8 g of licorice powder to mask the bitter taste of the proteins (~400 kJ, 68 energy percentage (E%) protein, 26 E% carbohydrate, 6 E% fat). Serving 2 consisted of a drink with 30 g hydrolyzed protein and 16 g licorice powder in 200 mL water and toast bread with mayonnaise, cucumber and bell peppers (3 MJ, 24 E% protein, 46 E% carbohydrate and 30 E% fat). Free tracer was added to the protein drinks to account for any dilution of infused tracer during the postprandial periods. The amount was calculated based on the expected enrichment obtained by infusion and according to the tracer content in the protein. The amino acid composition of the three proteins is presented in Table 1. 

### 2.5. Muscle Biopsies

Muscle biopsies were taken from *vastus lateralis quadriceps* using a 5 mm biopsy needle (Pelomi Medical, Denmark) with manual suction under sterile conditions and local anesthesia (3–5 mL 1–2% lidocaine). The order of the leg used (left/right/left or right/left/right) as well as the order of the placements of the biopsies in the muscle (top, middle and bottom) were randomly determined for all participants according to predefined randomization sequences. One incision was made for each biopsy and all three biopsies were taken from the same leg per test day. Muscle specimens were weighed to assure a minimum of 30 mg muscle. The biopsy was immediately washed in cold saline (NaCl concentration: 9 mg/mL) to remove blood and visible adipose tissue. The muscle specimen was immediately frozen in liquid nitrogen in a sealed container and stored at −80 °C until further analysis.

### 2.6. Blood Samples

Blood samples were collected using the heated hand box technique, to obtain arterialized venous blood [19]. The hand was placed in a cavity through which heated air circulates to warm the hand to 50 °C and blood is collected through an indwelling superficial catheter placed at the back of the hand. Blood samples were collected into EDTA tubes centrifuged for 10 min at 4 °C at 2900× *g* and stored at −80 °C until analysis for plasma (p) amino acid concentrations and tracer abundances. 

### 2.7. Analyses

#### 2.7.1. Tracer Enrichment

Amino acid concentrations and phenylalanine abundances in plasma samples were measured using liquid chromatography tandem mass-spectrometry (LC-MS/MS) as described elsewhere [21]. Briefly, to 100 µL plasma, 100 uL internal amino acid labelled standard were added, mixed and the amino acids were purified by the following process: First, acidification by addition of 1 mL 50% acetic acid and poured over resin columns (AG 50W-X8 resin; Bio-Rad Laboratories, Hercules, CA, USA), eluted with 2 × 1 mL 2 M NH_4_OH, and dried down under a stream of nitrogen. The purified amino acids were derivatized to yield phenylthiocarbamyl derivites, dried and re-dissolved in 100 uL of LC buffer B and 10 uL was injected for analysis on a triple stage quadrupole mass-spectromter, TSQ Vantage (Thermo Fisher Scientific, San Jose, CA, USA).

#### 2.7.2. Muscle Biopsies

Skeletal muscle specimens of 20–25 mg wet weight were added an internal standard (U-^13^C_6_-leucine), homogenized (FastPrep 24, MP Biomedicals, Santa Ana, CA, USA) in 1.5 mL ice-cold saline for 2 × 45 sec in vials containing two silicium-carbide crystals and eight lysing beads (Lysing Matrix D, MP Biomedicals, Santa Ana, CA, USA), spun (4 °C, 5500 g, 10 min), and the supernatant transferred into new vials, containing 1.5 mL of 100% acetic acid, which was then poured over resin columns as described above. The amino acids were then eluted with NH_4_OH and derivatized mixing *N*-methyl-*N*-(*tert*-butyldimethylsilyl) trifluoroacetamide (MTBSTFA) + 1% *tert*-butyl-dimethylchlorosilane (Regis Technologies, Morton Grove, IL, USA) and acetonitrile in a 1:1 ratio using 15 uL each. The MTBSTFA-derivatized phenylalanine (M and M + 6, tracer) and leucine (M and M + 6, internal standard) abundances were analyzed in a gas chromatograph (GC) (Trace 1310, Thermo Scientific; Milano, Italy), quadrupole-mass spectrometer (TSQ Quantum; Thermo Scientific, San Jose, CA, USA) operated in electron ionization mode. The muscle free leucine concentrations were calculated by estimating the tissue fluid volume (uL) to be 80% of the dry weight (mg). 

The myofibrillar protein fraction was isolated from the remaining muscle protein pellet by adding 1 mL of a homogenization buffer (0.02 M Tris, pH 7.4, 0.15 M NaCl, 2 mM EDTA, 2 mM EGTA, 0.5% Triton-X 100, and 0.25 M sucrose) and homogenized 2 × 45 sec and then incubated for 3 h at 4 °C. It was centrifuged (800× *g*, 4 °C, 20 min) and the supernatant was discarded. This step with the addition of 1 mL homogenization buffer was repeated once, leaving the homogenate for 30 min and again discarding the supernatant. To the pellet, 1.5 mL of high salt buffer (0.7 M KCl and 0.1 M Na_4_P_2_O_7_, pyrophosphate) was added, vortexed, and left overnight at 4 °C. The next morning, samples were vortexed and centrifuged (1600× *g*, 4 °C, 20 min) and the supernatant containing the myofibrillar protein fraction was transferred to new vials to which we added 3.45 mL (×2.3 vol) ice-cold 99% ethanol, vortexed, and left for 2 h at 4 °C. Thereafter, samples were spun (1600× *g*, 4 °C, 20 min) and the supernatant was discarded. The pellet, containing the myofibrillar protein fraction, was washed once with 1 mL 70% ethanol, vortexed, and centrifuged (1600× *g*, 4 °C, 20 min). The myofibrillar proteins were hydrolyzed in 1 mL 6 M HCl at 110 °C overnight, after which the hydrolysates were diluted with 4 mL water and the constituent amino acids purified over resin columns (AG 50W-X8 resin; Bio-Rad Laboratories, Hercules, CA, USA) as described for the plasma amino acids. Hereafter, the amino acids were derivatized to the *N*-acetyl *n*-propyl derivatives, as described in detail previously [22]. The analysis of ^13^C-phenylalanine bound in myofibrillar proteins was performed on a gas chromatography-combustion (GC-C) isotope ratio mass Spectrometry (IRMS) system (Hewlett Packard 5890-Finnigan GC combustion III-Finnigan Deltaplus; Finnigan MAT; Bremen, Germany) [23].

#### 2.7.3. Other Biochemical Analyses

Urine samples were collected after both servings for analysis of postprandial urea concentrations, which were determined by ABX pentra Urea CP (Pentra 400 analyzers, Horiba ABX, Montpellier, France) with intra-CV: 2.0%, inter-CV: 4.0%. 

### 2.8. Calculations

Tracer-to-tracee (TTR) enrichment was determined by subtracting the isotope ratio of a background sample from the isotope ratios measured for the samples obtained during the infusion. The abundance of ^13^C-phenylalanine in myofibrillar proteins was converted to TTR from the ∂-value measured by the IRMS analysis by the following formula:

0.0112372 × (0.001 × ∂ + 1)(1)

Myofibrillar fractional synthesis rate (FSR) was calculated using the standard precursor-product model based on the incorporation of [*ring*-^13^C_6_] phenylalanine into myofibrillar proteins:

FSR (%/h) = ∆E_protein_/(E_precursor_ × ∆time(h)) × 100% (2)

where ∆Eprotein is the difference in tracer enrichment in the myofibrillar proteins between two adjacent muscle biopsies. Eprecursor is the weighted average enrichment of [*ring*-^13^C_6_] phenylalanine in the muscle free pool during the incorporation time and ∆time is the time between two biopsies. 

Muscle free leucine concentrations (c_leu_) were calculated from:

c_leu_ = ((c_IS_ × vol_IS_)/(ratio (M + 6)/M)) × vol_muscle_ (3)

where c_IS_ and vol_IS_ are the concentration and the volume, respectively, of the internal standard (IS) (U-^13^C_9_-leucine) used. vol_muscle_ is the watery pool in μL of a muscle specimen estimated as 80% of the muscle wet weight in mg.

Area under the curve (AUC) were calculated for p-leucine, p-phenylalanine, p-branched-chain amino acids (BCAA), p-EAA, and p-total amino acids after serving 1 (0 (baseline)–150 min) and serving 2 (150–310 min) using the trapezoidal rule. Data on EAA do not include BCAA and data on EAA and total amino acids do not include phenylalanine as this was infused in a large amount in the prime and hence, does not reflect protein intake.

#### 2.8.1. Sample Size 

The sample size was based on a power calculation where the least detectable difference in FSR was set to 0.01%/h and the within subject standard deviation to 0.01%/h. With a power of 0.8 and a significance level of 0.05 a total of 18 completers was required. 

#### 2.8.2. Statistical Analyses

Differences in FSR between proteins after consumption of serving 1 or serving 2 were analyzed using a linear mixed-effects model with a serving-treatment interaction, treatment order, and age as fixed effects and with subject-specific random effects. Model reduction was carried out by first testing the interaction term, and, if non-significant, the main effects of serving and treatment were evaluated subsequently. The treatment main effect can be seen as accumulated effects, e.g., comparing the overall FSRs for both serving periods between the three protein types. Pairwise comparisons were reported where appropriate.

Moreover, concentrations of p-leucine, p-phenylalanine, p-BCAA, p-EAA, p-total amino acids, intramuscular free leucine concentrations and intramuscular and plasma tracer enrichments, urine urea concentrations, as well as p-leucine and p-phenylalanine AUC_serving1_ and AUC_serving2_ were analyzed as using a linear mixed-effects model with a serving-treatment interaction and with treatment order, and age as fixed effects and with subject-specific random effects. Analysis on intramuscular free leucine was adjusted for baseline value. As for the FSRs, tests for interaction and main effects were carried out.

Time to peak for p-leucine concentrations were determined as the time point at which the highest concentration was seen after consumption of both serving 1 and 2, respectively. P-leucine concentration time to peak was tested after serving 1 and 2 individually using a linear mixed-effects model with a treatment effect and age as fixed effects and with subject-specific random effects.

All data are presented as means and standard error of means (SEM), unless otherwise specified. Before statistical analyses were conducted, all data were checked for normality and homogeneity of variance. If data were not normally distributed, they were transformed according to best fitted transformation.

Statistical analyses were performed using STATA version 13.1 (StataCorp. 2013, StataCorp LP, College Station, TX, USA). 

## 3. Results

### 3.1. Subject Characteristics

Eighteen subjects completed the study (Table 2). While conducting the trials, two included subjects dropped out between the screening visit and initiation of the study as they could not fit the experimental visits into their schedules and one subject dropped out after his first visit due to illness. Therefore, three more were recruited to end at *n* = 18 completers.

### 3.2. Phenylalanine Concentration and Enrichment in Plasma and Muscle

**Figure 2:**
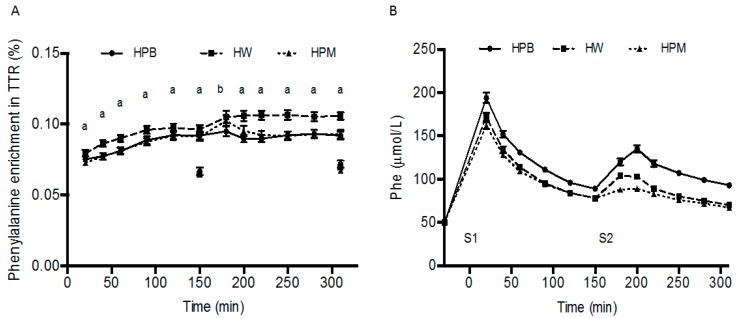
Mean ± SEM tracer enrichments (tracer-to-tracee ratio (TTR)) in plasma (p) (dots with lines) and muscle (dots at 150 and 310 min only) following the flood-primed continuous infusion with [*ring*-^13^C_6_] phenylalanine (**A**), and p-phenylalanine concentrations after consumption of serving 1 and serving 2 (**B**), *n* = 18. (**A**) ^a^ HW significantly different from HPB and HPM, *p <* 0.05. ^b^ HPB significantly different from HPM, *p <* 0.05. Data on TTR were analyzed using linear mixed-effects models with a time-treatment interaction and treatment order and age as fixed effects and with subject-specific random effects. Data on p-phenylalanine were analyzed as area under the curve (AUC): AUC_serving1_ and AUC_serving2_ using a linear mixed-effects model with a serving-treatment interaction and with treatment order and age as fixed effects and with subject-specific random effects. HPB, hydrolyzed porcine blood protein; HPM, hydrolyzed porcine muscle protein; HW, hydrolyzed whey protein.

The intravenous injection of the flood prime resulted in a marked increase in phenylalanine concentration and also introduced the tracer immediately in the arterialized blood in an enrichment (7.6% at 20 min after injection (mean of all groups)), which was maintained by the continuous infusion (Figure 2). P-phenylalanine enrichment did though differ between proteins (*p* < 0.0001). It was higher at all time-points for HW than for HPB and HPM (except at 180 min) (all *p* < 0.05) and did not differ between HPB and HPM except at time 180 min, where it was higher for HPM than HPB (*p* < 0.001). AUC_serving1_ and AUC_serving2_ for p-phenylalanine concentrations were higher for HPB (serving 1: 18239 ± 345 µmol/L × 150 min, serving 2: 17593 ± 445 µmol/L × 160 min) than HW (serving 1: 15978 ± 381 µmol/L × 150min, serving 2: 13542 ± 185 µmol/L × 160min, *p* < 0.001) and HPM (serving 1: 15518 ± 312 µmol/L × 150min, serving 2: 12512 ± 158 µmol/L × 160min, *p* < 0.001). AUC_serving1_ for p-phenylalanine concentrations was similar for HW than HPM (*p* = 0.10) and AUC_serving2_ was higher for HW than HPM (*p* < 0.001) (Figure 2). The tracer enrichment in the intramuscular pool was on average 71% of the enrichment in arterialized plasma at the corresponding time points, with no difference between proteins (*p* = 0.36), but a slightly higher enrichment for all proteins at 310 min compared to 150 min *(p* < 0.05) (Figure 2).

### 3.3. Myofibrillar Protein Synthesis

**Figure 3:**
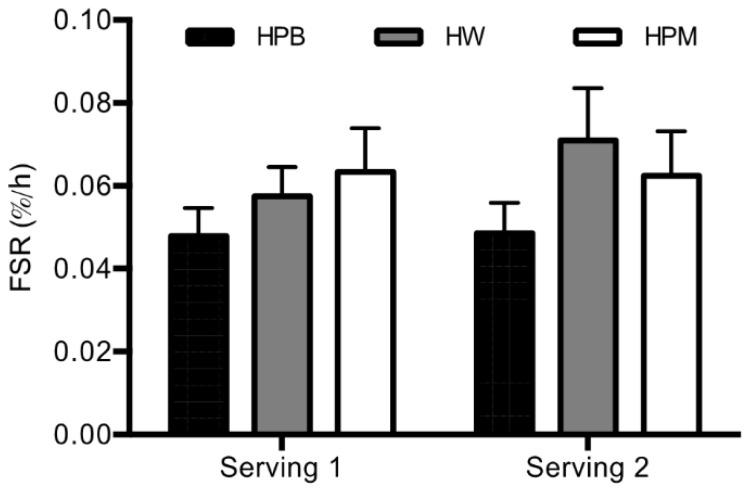
Mean ± SEM 2.5 h FSR after serving 1 and serving 2, *n* = 18. Differences in FSR between proteins were analyzed using a linear mixed-effects model with a serving-treatment interaction and with treatment order and age as fixed effects and with subject-specific random effects. There was no effect of protein or serving (*p ≥* 0.14). FSR, fractional synthesis rate; HPB, hydrolyzed porcine blood protein; HPM, hydrolyzed porcine muscle protein; HW, hydrolyzed whey protein.

The mixed-effects model revealed no serving-protein type interaction (*p* = 0.66), and the subsequent model reduction revealed no effect of the main effects, serving (*p* = 0.53) and protein type (*p* = 0.14). Hence, over all muscle FSR neither differed between servings (15 g protein alone or 30 g in a mixed meal), nor protein types (Figure 3). However, due to the nature of the protein type FSR values we report the pairwise comparisons of protein types for pooled servings and HPB tended to be lower than HW and HPM (*p* = 0.067 and *p* = 0.093, respectively), while no difference was apparent between HW and HPM (*p* = 0.88).

### 3.4. Plasma Amino Acids and Muscle Free Leucine Concentrations

**Figure 4:**
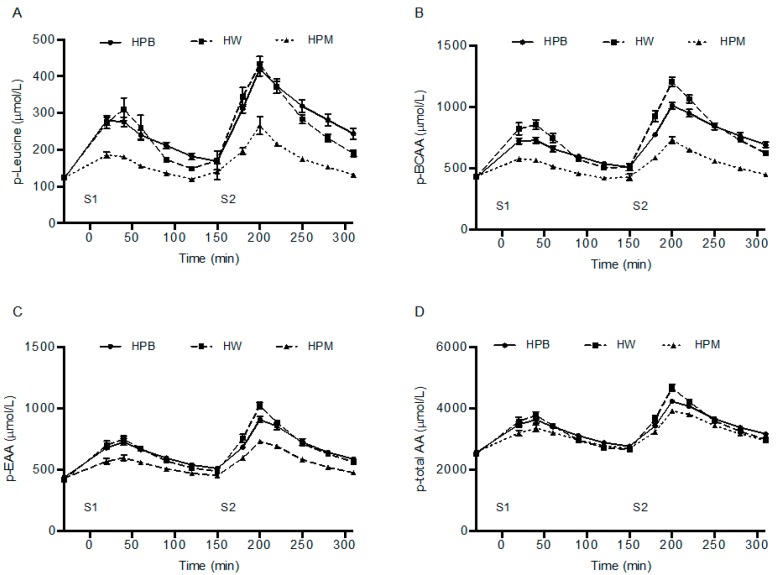
Mean ± SEM postprandial p-leucine (**A**), p-BCAA (**B**), p-EAA (**C**) and p-total amino acid (**D**) concentrations after serving 1 and serving 2, *n* = 18. Time to peak and peak p-leucine concentrations were analyzed using linear mixed-effects models with treatment order, age and baseline p-leucine concentrations as fixed effects and with subject-specific random effects. Data on p-leucine, p-BCAA, p-EAA and p-total amino acids were analyzed as AUC_serving1_ and AUC_serving2_ using a linear mixed-effects model with a serving-treatment interaction and with treatment order and age as fixed effects and with subject-specific random effects. HPB, hydrolyzed porcine blood protein; HPM, hydrolyzed porcine muscle protein; HW, hydrolyzed whey protein, S1, serving 1 (15 g protein); S2, serving 2 (30 g protein served with a mixed meal).

P-leucine concentrations increased for all proteins after both servings (Figure 4). After consumption of serving 1 and serving 2 peak p-leucine concentrations were higher for HW and HPB than HPM (*p* < 0.001), with no difference between HW and HPB (*p ≥* 0.28). By using the time for when leucine concentration was highest within the groups we compared the time to peak, although this is not visual from Figure 4. After consumption of serving 1 there was no difference in time to peak between proteins (*p* = 0.16), whereas peak p-leucine concentration was reached more rapidly after HW than HPB (*p* < 0.01) and tended to be reached more rapidly after HW than HPM (*p* = 0.059) after consumption of serving 2. No difference was found between HPM and HPB (*p* = 0.32).

AUC for amino acids were calculated after each of the servings. AUCs after serving 1 were: p-leucine, HPB = HW > HPM; p-BCAA, HW > HPB > HPM; p-EAA, HPB = HW > HPM; p-total amino acids, HPB = HW > HPM. AUCs after serving 2 were: p-leucine HPB ≥ HW > HPM; p-BCAA, HW = HPB > HPM; p-EAA, HPB = HW > HPM; p-total amino acids, HPB = HW > HPM (Figure 4). 

**Figure 5:**
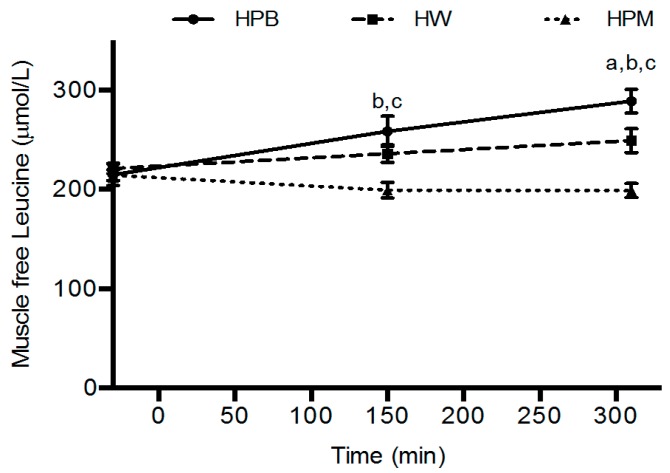
Mean ± SEM muscle-free leucine concentrations, *n* = 18. ^a^ HPB significantly different from HW, *p* < 0.01. ^b^ HPB significantly different from HPM, *p* < 0.01. ^c^ HW significantly different from HPM, *p* < 0.01. Data were analyzed using linear mixed-effects models with a time-treatment interaction and treatment order, age and muscle-free leucine concentrations at baseline as fixed effects and with subject-specific random effects. HPB, hydrolyzed porcine blood protein; HPM, hydrolyzed porcine muscle protein; HW, hydrolyzed whey protein.

Muscle free leucine concentrations were higher for HPB and HW than HPM (*p* < 0.01) at 150 min, with no difference between HPB and HW (*p* = 0.08). At 310 min muscle free leucine concentrations were higher for HPB than HW (*p* < 0.01) and HPM (*p* < 0.001), and higher for HW than HPM (*p* < 0.001) (Figure 5).

### 3.5. Urea

**Figure 6:**
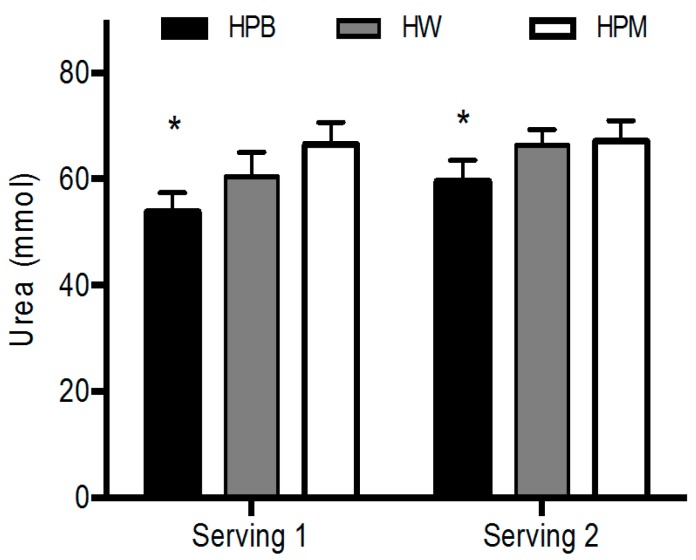
Mean ± SEM postprandial urea concentrations after serving 1 and serving 2, *n* = 18. * HPB significantly different from HPM and HW, *p* < 0.01. Data were analyzed using a linear mixed-effects model with a serving-treatment interaction and treatment order, age and as fixed effects and with subject-specific random effects. HPB, hydrolyzed porcine blood protein; HPM, hydrolyzed porcine muscle protein; HW, hydrolyzed whey protein

Postprandial urea concentrations were lower for HPB than HPM and HW after serving 1 (*p* < 0.05) and after serving 2 (*p* < 0.01) with no significant effect of serving. There was no difference between HW and HPM (*p ≥* 0.44) (Figure 6).

## 4. Discussion

The primary finding in the present study is that hydrolyzed porcine proteins from blood and muscle result in an MPS not significantly different from hydrolyzed whey protein when consumed in a moderate dose alone (15 g) and in a dose of 30 g as part of a mixed meal. However, post hoc comparisons revealed that MPS after intake of blood protein tends to be lower than after intake of both porcine muscle and whey suggesting an inferior effect of porcine blood. Further, no difference in MPS was apparent between servings meaning that muscle protein synthesis rate is stimulated equally by 15 g protein alone and 30 g in a mixed meal. 

The anabolic effect of whey protein has been extensively studied and its immediate stimulatory effect on MPS is yet to be surpassed by other proteins [13]. The present study is the first to investigate the anabolic effect of hydrolyzed porcine proteins originating from muscle and blood and comparing it with hydrolyzed whey protein on human skeletal muscle. Previous studies on whey protein have primarily examined intact protein [4,5,9,24], therefore the use of whey protein hydrolysate in the present study will be briefly discussed. Intact whey protein is quickly digested hence its constituent amino acids are readily absorbed in the gut peaking in the circulation within the first hour after intake [5]. Due to this characteristic of whey, the impact of hydrolysis appears somehow minor [25,26], while hydrolysis of slower-digestible proteins like casein markedly impacts the absorption rate [25,26]. To our knowledge, the effect of intact versus hydrolyzed whey protein on MPS has not been studied in humans. Only 15 g intact whey protein was found to be superior to 7 g EAA, which corresponded to the EAA content in the 15 g intact whey protein [27]. This finding is supported by rodent data showing that a whey protein hydrolysate was superior to a mixture of amino acids with the exact same profile as whey protein to stimulate MPS [28]. Therefore, we argue that whey protein may contain proteins/peptides that favors an amino acid appearance profile that is superior to that of the single amino acids’ appearance carries and therefore that whey hydrolysate is a valid positive control in the present study for comparison with the porcine proteins. 

The procedures to isolate target protein fractions from porcine muscle and blood including enzymatic hydrolysis eliminated major differences in absorption rate between proteins shown as rapid appearance of amino acids in arterialized blood from all three protein sources (Figure 4 and time to peak results). However, the amino acid composition of the three protein sources diverged (Table 1) affecting the postprandial circulating amino acid concentrations accordingly. Hence, amino acid concentrations were lower for hydrolyzed porcine muscle protein than hydrolyzed porcine blood protein and hydrolyzed whey protein (Figure 4). Despite these rather marked compositional differences and resulting AUCs for leucine, BCAA and EAA, the amino acid contents in 15 g hydrolyzed porcine muscle protein did result in an even 3 h postprandial MPS (Figure 3, serving 1). The reason may be that in the resting muscle of healthy adult men, 15 g quickly accessible high quality protein may make up an optimal dose. It could be that prior exercise could have created a condition where 15 g was sub-optimal, as the dose-response relationship between protein intake and MPS appears right-shifted (larger dose required to maximal MPS stimulation) [3,4,29]. However, post-exercise recovery was not the purpose of this study and we wanted also to compare the 3 h postprandial periods directly after the two serving sizes. 

Also of interest, we showed that in young male subjects the MPS response was not significantly impacted by doubling the protein amount from 15 to 30 g and adding energy as part of a mixed meal (Figure 3). First of all this underlines the fact that the MPS might have been stimulated close-to-maximal already with the 15 g serving. Further, despite no impact of protein serving sizes and energy intake on MPS, should be acknowledged that the leg muscle protein net balance presumably was improved by serving 2 compared to serving 1. Recently, a linear association was shown between energy/protein content and whole body protein net balance [8]. Insulin may play a role in the net balance improvement after full energy meals. Hyperinsulinemia (intake of carbohydrate) does not have an additive impact of MPS beyond that of protein [30,31] but it must be expected that the anticipated impact of energy intake on insulin secretion in the present study most likely has had a major impact on the protein breakdown rate and hence, the net protein balance. We did neither measure leg protein breakdown nor net protein balance and therefore will not discuss this balance further as it remains speculative. The speed of the translational apparatus appears to be driven largely by the availability and the pattern of hyperaminoacidemia in the circulation [5]. As the FSR approach measures a gross average of the synthesis rate over a given period of time and misses temporary fluctuations and peaks, we cannot rule out that larger rates and divergent rates between protein sources may have been present within the 150-min time frames as the peak amino acid concentrations appeared before 60 min after serving 1 and around 60 min after serving 2. However, any such effect may be ascribed as being minor. 

More specifically, the statistical analysis of the comparison of MPS rates pooled across servings revealed a tendency toward a lower FSR after intake of hydrolyzed porcine blood protein compared to hydrolyzed whey and porcine proteins (*p* = 0.067 and *p* = 0.093, respectively).Both of these results support the notion that hydrolyzed porcine blood protein may be slightly inferior to the other two proteins. However, the differences did not reach statistical significance despite the rather high power in the present study (*n* = 18 in cross-over investigations, see limitation section) and hence, any MPS stimulatory differences between protein sources must be concluded as being minor. 

Intramuscular concentrations of amino acids are utilized for protein synthesis or metabolized quickly once they appear and only seems to accumulate with high circulating concentrations [32] and therefore, the intramuscular concentrations most likely are beyond any accumulation and reflect levels approaching baseline values (Figure 5). A comparison with the plasma leucine concentrations at the time points for muscle biopsies reveals a concordance in the concentration differences between groups. The porcine muscle protein contains least leucine and appears lowest in both plasma and muscle. However, after intake of porcine blood hydrolysate the plasma concentrations remained elevated towards the end, 90 min and 280 min, of the postprandial periods after serving 1 and 2, respectively, which reflects either a prolonged appearance from gut or a slower clearance from circulation. Interestingly, also the intramuscular concentrations are higher after hydrolyzed porcine blood protein intake than after whey. We are not aware of any similar findings, however, the results suggest that intracellular utilization, for protein synthesis (trend toward a slower rate compared to whey) and/or metabolism/oxidation (a lower urea concentration) is diminished after intake of hydrolyzed porcine blood protein compared to muscle protein and whey.

This study has a number of limitations. The lack of a fasting period means that we cannot say whether intake of any of the protein intakes enhanced the MPS over that of a basal rate. This design was chosen due to ethical concerns with number of muscle biopsies. Furthermore, the statistical comparisons made were simpler and more powerful with fewer time points. We argue though, that sufficient evidence support that whey protein stimulates muscle protein synthesis above fasting level [3,9]. Given the borderline finding of HBP being lower than HW and HBM, it can be argued that the study was underpowered to detect an actual difference. However, an *n* of 18 inclusions in a cross-over design with the application of the tracer methodology has high power. In contrary, the repeated tracer exposures may have enhanced variation in the FSR measurements randomly [33] and thereby diminished the benefits of the cross-over design, comparably. Finally, with reference to the ‘muscle full’ phenomenon, it could be argued that the resting muscle in our subjects entered a kind of refractory period with serving 2 only 2.5 h after ingestion of serving 1 [34,35] and therefore, a dampened stimulatory response was seen after serving 2. The pulsatile circulating amino acid concentrations after serving 1 should though, allow the muscle to sense the lack of excess availability and thereby, be responsive for yet another meal. 

## 5. Conclusions

In conclusion, 15 g of hydrolyzed protein alone or 30 g ingested as part of a mixed meal result in similar muscle FSRs in young resting males, emphasizing the notion that proteins/amino acids are primarily responsible for stimulating the MPS and that this is maximized irrespective of other nutrients and concomitant energy intake. Further, the data support that the postprandial protein net balance cannot be extrapolated from the synthesis rates. When ingested alone and as part of a mixed meal, intake of hydrolyzed porcine proteins from blood tends to result in a lower MPS in the muscle of young males in the resting condition than intake of porcine muscle and whey protein hydrolysates. All in all, these findings lend support for the use of especially hydrolyzed porcine muscle-derived proteins as well as whey proteins as effective sources for stand-alone supplements or ingredients in snacks and meals to support growth and maintain mass of skeletal muscle. However, investigations involving the measurement of protein breakdown rate and/or net balance over the course of multiple servings comparing different protein sources should be conducted to reveal any differences in their net anabolic potentials when integrated as parts of a full diet. 

## Figures and Tables

**Table 1:** Amino acid (AA) composition in 15 g protein.

	HW	HPB	HPM
	g AA/15 g protein		
Alanine	0.8	1.3	1.0
Arginine	0.4	0.6	1.1
Aspartic acid	1.7	1.7	1.1
Cystein + cysteine	0.4	0.1	0.1
Glutamic acid	2.7	1.2	2.3
Glycine	0.3	0.8	1.3
Histidine	0.3	1.1	0.4
Isoleucine	1.0	0.1	0.4
Leucine	1.7	1.9	0.8
Lysine	1.4	1.5	1.0
Methionine	0.3	0.1	0.3
Phenylalanine	0.5	1.0	0.3
Proline	0.9	0.5	0.9
Serine	0.8	0.7	0.4
Threonine	1.1	0.6	0.4
Tryptophan	0.3	0.3	0.1
Tyrosine	0.5	0.4	0.3
Valine	0.9	1.2	0.5
EAA	6.1	6.2	3.1
BCAA	3.6	3.2	1.7
Total AA ^1^	16.0	15.1	12.7

Data are presented as g AA/15 g protein. ^1^ Total AA differs from 15 g protein due to different analyses for total N and specific AA. AA, amino acids; BCAA, branched-chain amino acids; EAA, essential amino acids; HPB, hydrolyzed porcine blood protein; HPM, hydrolyzed porcine muscle protein; HW, hydrolyzed whey protein.

**Table 2:** Subject characteristics of completers at baseline.

Characteristics	
Age (years)	24 ± 1
Body weight (kg)	74.5 ± 1.5
Height (cm)	185.5 ± 1.3
BMI (kg/m^2^)	21.7 ± 0.4
Fat-free mass (kg) ^1^	62.6 ± 1.0
Fat mass (kg) ^1^	13.6 ± 1.2

Data are presented as mean ± standard deviation (SD), *n* = 18. ^1^ Body composition estimated by dual-energy X-ray absorptiometry.

## Abbreviations

AUC	area under the curve
BCAA	branched-chain amino acids
DXA	Dual-energy X-ray absorptiometry
E%	energy percentage
EAA	essential amino acids
FSR	fractional synthesis rate
GC-C	gas chromatography-combustion
HPB	hydrolyzed porcine blood protein
HPM	hydrolyzed porcine muscle protein
HW	hydrolyzed whey protein
IRMS	isotope ratio mass Spectrometry
LC-MS/MS	liquid chromatography tandem mass-spectrometry
MPS	muscle protein synthesis
p	plasma
s	serum
TTR	tracer-to-tracee ratio
